# Positive Regulation of Soybean Seed Vigor and Quality by the Zinc Finger Transcription Factor GmPHD3 Under High-Temperature and High-Humidity Stress

**DOI:** 10.3390/plants15152376

**Published:** 2026-08-03

**Authors:** Yangyang Zhao, Tianle Li, Jing Chen, Zhiqin Xue, Yuehua Yu, Lili Zhang, Ruoxi Li, Wei Su, Hang Shen, Lifang Zhuang, Hao Ma

**Affiliations:** 1 State Key Laboratory of Crop Genetics & Germplasm Enhancement and Utilization, Nanjing Agricultural University, Nanjing 211899, China; 2 College of Agronomy, Xinjiang Agricultural University, Urumqi 830052, Chinazlili9965@126.com (L.Z.)

**Keywords:** seed germination, abiotic stress response, zinc finger transcription factor, germination rate, soybean quality

## Abstract

Field high-temperature and high-humidity (HTH) stress causes soybean seed deterioration, including shrinkage, moldiness, browning, and reduced germination rate, thereby decreasing seed vigor, emergence performance, and commercial value. Clarifying the molecular mechanisms of seed vigor formation under HTH stress is critical for identifying key genes and improving spring soybean seed quality. Plant homeodomain (PHD) proteins are conserved zinc-finger transcription factors involved in chromatin remodeling and stress responses. GmPHD3, a soybean PHD family member, has only been studied via heterologous expression in Arabidopsis; its authentic function in soybean remains unclear. In this study, we functionally characterized GmPHD3 in soybean. GmPHD3 is a nuclear-localized transcription factor containing Alfin and PHD domains, and is evolutionarily conserved across legumes and other plant species. Expression analysis showed that *GmPHD3* was strongly induced by HTH stress, with rapid induction at 6 h in the tolerant cultivar Xiangdou No. 3 but delayed induction at 48 h in the sensitive Ningzhen No. 1. The *GmPHD3* promoter harbors abscisic acid (ABA) and stress-responsive elements, and GUS assays confirmed its response to ABA and HTH. Overexpression of *GmPHD3* in soybean significantly enhanced seed vigor under artificial accelerated aging by increasing the germination speed and activities of SOD, POD, while reducing TBARS content. Meanwhile, *GmPHD3* negatively regulated seed longitudinal elongation, leading to a short and round seed shape without changing single-seed weight. It also significantly decreased the proportion of palmitic acid, a major saturated fatty acid. These results demonstrate that *GmPHD3* positively regulates soybean seed vigor and stress resistance under HTH stress by modulating the antioxidant system and modifying seed morphology and fatty acid composition, providing a valuable target for soybean molecular breeding.

## 1. Introduction

Plant homeodomain (PHD) proteins are a class of conserved zinc-finger transcriptional regulators widely distributed in eukaryotes, and their conserved PHD domains are core functional units involved in chromatin remodeling and transcriptional regulation [1]. The PHD domain consists of approximately 60 amino acid residues and possesses a typical Cys4-His-Cys3 zinc-binding motif, which is highly conserved during species evolution and exists in hundreds of eukaryotic proteins to perform fundamental biological functions [2,3]. Plant PHD proteins exhibit diverse structural architectures. In addition to the conserved core PHD domain, family members contain various auxiliary functional domains, forming distinct structural combinations that contribute to functional differentiation [4]. Several domain architectures are evolutionarily conserved across plant lineages, and these conserved structures are closely associated with the essential biological functions of PHD proteins [5,6]. The variable domain composition enables PHD proteins to participate in transcriptional regulation, protein–protein interaction, and epigenetic modification recognition, laying a molecular foundation for their diverse biological functions [7,8].

PHD family proteins participate in the regulation of multiple plant growth, developmental, and stress response processes. Extensive studies have demonstrated that PHD genes play vital roles in floral transition, meiosis, pollen development, organ differentiation, and seed development in various plant species [9,10,11,12,13,14,15,16,17]. In addition, PHD proteins are important regulators of plant abiotic stress tolerance and are widely involved in the response to high temperature, high humidity, salt, drought, and heavy metal stresses [18]. Stress-induced expression changes of PHD genes enable plants to activate downstream signaling pathways and enhance stress adaptation [19]. In soybean, numerous GmPHD family members exhibit differential expression under salt, alkali, and drought stresses, indicating their extensive functions in abiotic stress regulation [19]. Although previous studies have reported the stress tolerance functions of several GmPHD members, the roles of PHD proteins in regulating soybean seed tolerance to high temperature and high humidity (HTH) stress remain largely unexplored.

HTH stress commonly occurs during the maturation and postharvest storage stages of soybean in southern China, which easily induces seed deterioration, reduces seed vigor and germination rate, and ultimately deteriorates seed quality and affects safe seed storage and high-quality soybean production. Clarifying the molecular mechanism underlying soybean seed HTH tolerance is of great practical significance for breeding high-vigor and stress-tolerant soybean cultivars. Our previous studies preliminarily identified a HTH-responsive transcription factor, GmPHD3, which interacts with the key HTH stress regulator GmSBH1 and exhibits distinct expression patterns in soybean cultivars with different HTH tolerance, implying its potential regulatory function in the soybean HTH stress response.

Nevertheless, the current functional understanding of GmPHD3 is only based on heterologous expression in Arabidopsis, which cannot accurately reflect its endogenous function in soybean. Whether *GmPHD3* regulates soybean seed vigor formation under HTH stress and its underlying molecular mechanism remain unclear. Therefore, the present study constructed *GmPHD3* overexpressing soybean materials and performed systematic molecular and physiological analyses. The objectives were to elucidate the molecular mechanism by which *GmPHD3* regulates soybean seed vigor under HTH stress and to provide candidate gene resources and a theoretical basis for the molecular breeding of high-vigor soybean.

## 2. Results

### 2.1. Bioinformatics Analysis and Subcellular Localization Prediction of the GmPHD3 Gene

**Figure 1:**
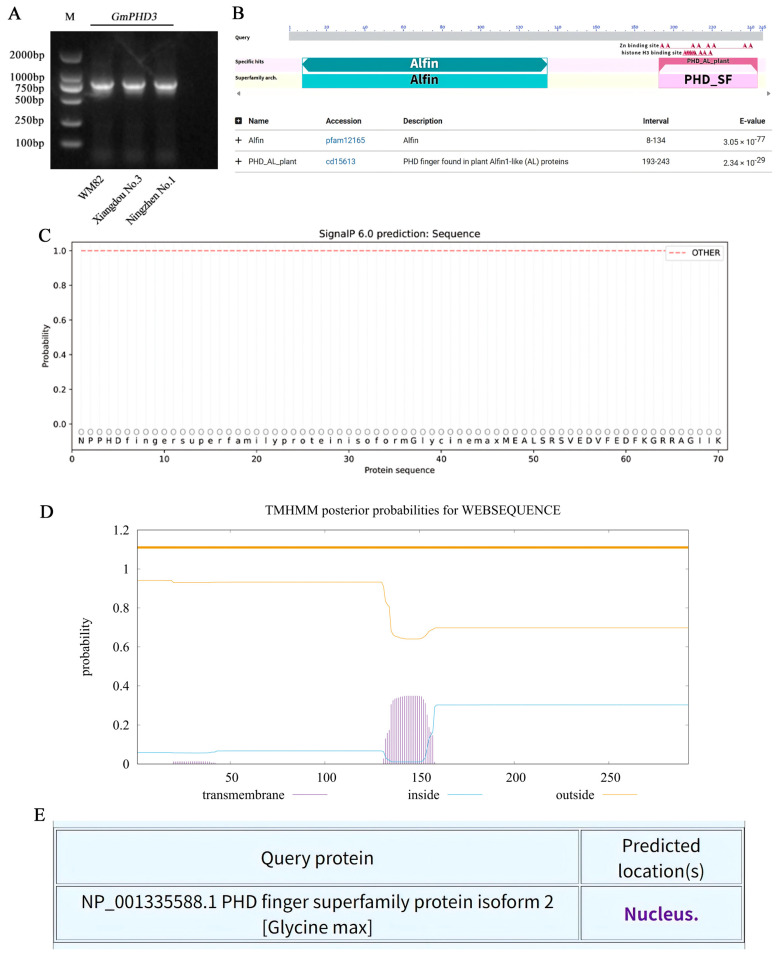
Cloning and bioinformatics analysis of soybean *GmPHD3*. (**A**) Agarose gel electrophoresis of *GmPHD3* PCR amplification products. (**B**) Conserved domain analysis of the GmPHD3 protein, containing an N-terminal Alfin domain and a C-terminal PHD-type zinc finger domain. (**C**) SignalP 6.0 prediction indicating no signal peptide in GmPHD3. (**D**) TMHMM 2.0 prediction showing no transmembrane domain in GmPHD3. (**E**) ProtComp 9.0 prediction indicating nuclear localization of GmPHD3.

The *GmPHD3* gene (LOC100170696) was cloned from Xiangdou No. 3 and Ningzhen No. 1 (Figure 1A). Sequencing results showed that the CDS sequences from both cultivars were completely consistent with the reference genome. NCBI CDD analysis indicated that GmPHD3 contains two highly conserved functional domains (Figure 1B): an N-terminal Alfin domain, which is a characteristic feature of ALFIN-like transcription factors and is predicted to participate in the specific recognition of DNA *cis*-acting elements; and a C-terminal plant-specific PHD-type zinc finger domain (PHD-AL-plant) harboring conserved zinc-binding and histone H3-binding sites, implying that this protein may be involved in chromatin remodeling and epigenetic regulation by recognizing histone modifications. The synergistic function of these two domains provides a molecular structural basis for GmPHD3 to act as a nuclear-localized transcription factor.

The full-length CDS of *GmPHD3* is 738 bp, encoding 246 amino acids. Physicochemical property prediction using Expasy showed that the protein has a molecular weight of 27.92 kDa and a theoretical isoelectric point (pI) of 5.08, indicating a weakly acidic protein. It contains 43 negatively charged residues (Asp + Glu) and 32 positively charged residues (Arg + Lys). SignalP-6.0 analysis revealed that GmPHD3 has no typical N-terminal secretory signal peptide (Sec/SPI type). The prediction probabilities of signal peptide (SP) and cleavage site (CS) were close to 0, while the probability of the non-signal peptide region (OTHER) remained approximately 1.0, confirming the absence of a signal peptide (Figure 1C).

TMHMM 2.0 prediction showed no typical transmembrane helices (TMH) in GmPHD3. The transmembrane probability curve was below 0.5 along the entire sequence, suggesting that the protein lacks the structural feature of transmembrane insertion, which is consistent with the characteristics of a soluble nuclear protein (Figure 1D). Subcellular localization prediction using Plant-mPLoc indicated that GmPHD3 is localized in the nucleus, further verifying its identity as a nuclear-localized transcription factor (Figure 1E).

**Figure 2:**
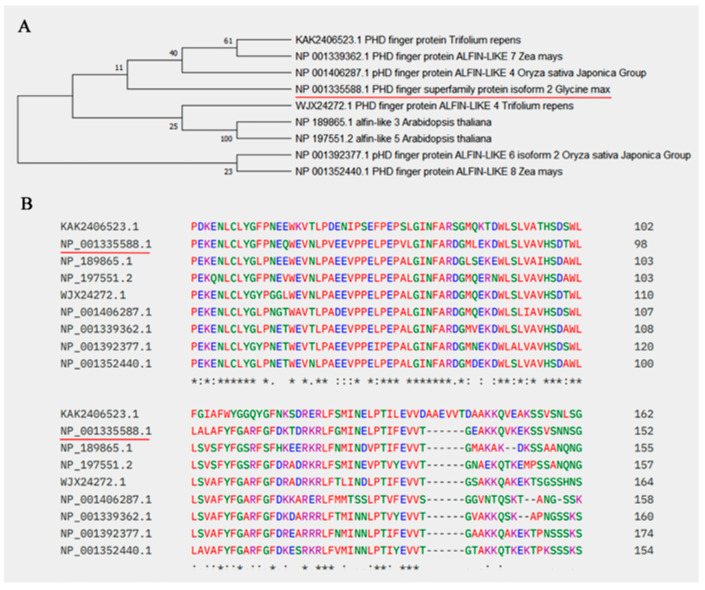
Phylogenetic and conserved domain analysis of GmPHD3. (**A**) Neighbor-Joining phylogenetic tree of GmPHD3 and its homologous proteins constructed using MEGA X. GmPHD3 exhibits the closest evolutionary relationship with homologous proteins from the legume species Trifolium repens. The sequence of GmPHD3 is marked with a red underline. (**B**) Multiple sequence alignment of GmPHD3 with ALFIN-like family homologous proteins from different plant species performed by Clustal Omega. The core sequences of the N-terminal Alfin domain and the C-terminal PHD-type zinc finger domain are highly conserved. Asterisks (*) indicate fully conserved residues, colons (:) indicate residues with strongly similar physicochemical properties, and dots (.) indicate residues with weakly similar physicochemical properties.

To analyze the evolutionary relationship of GmPHD3, homologous amino acid sequences of its ALFIN-like family proteins from *Arabidopsis thaliana*, *Oryza sativa*, *Zea mays* and *Trifolium repens* were retrieved via NCBI Blastp. A phylogenetic tree was constructed using the Neighbor-Joining (NJ) method in MEGA X. The results showed that GmPHD3 (NP 001335588.1) clustered closely with Trifolium repens (KAK2406523.1), rice PHD4 (NP 001406287.1) and maize PHD7 (NP 001339362.1), indicating their close genetic relationship. Distinct clades formed by homologs from Arabidopsis, rice, and maize revealed species-specific evolution of plant ALFIN-like transcription factors (Figure 2A).

To assess sequence conservation, multiple sequence alignment between GmPHD3 and eight homologous proteins was performed using Clustal Omega. The results showed high overall conservation, with highly consistent amino acid residues in the core functional regions. The N-terminal region contained consecutive fully and strongly conserved residues (marked by * and :), which constituted the core sequence of the Alfin domain. The C-terminal region was also highly conserved and harbored the characteristic motifs of the PHD-type zinc finger domain. Key sequences such as WICCD and FHGKC, as well as the zinc-binding and histone H3-binding residues, were nearly completely conserved across all aligned sequences. Only the linker region between the two domains and partial sequences at the N- and C-termini showed minor amino acid substitutions, deletions, or insertions, and none of these variations occurred at functional sites (Figure 2B).

**Figure 3:**
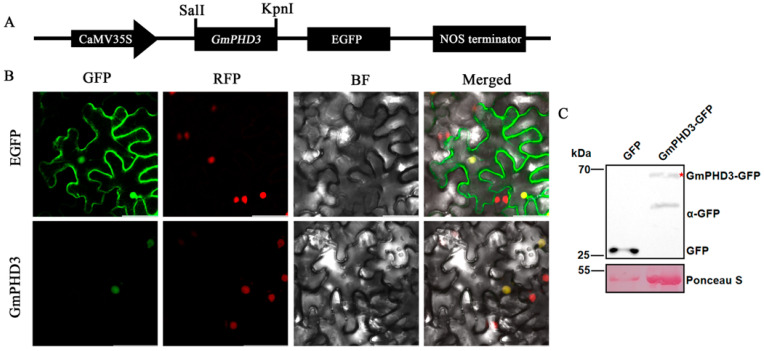
Subcellular localization and fusion protein integrity analysis of GmPHD3. (**A**) Schematic diagram of the CaMV 35S promoter-driven GmPHD3-EGFP fusion expression vector. Restriction enzyme sites (SalI, KpnI) used for cloning are indicated. (**B**) Subcellular localization of GmPHD3 in *Nicotiana benthamiana* leaf epidermal cells. GmPHD3-EGFP (bottom row) or the empty EGFP control (top row) was co-expressed with the nuclear marker H2B-RFP. GFP channel shows green fluorescence; RFP channel shows red fluorescence of the nuclear marker; BF, bright-field; Merged, overlay of all channels. Scale bar: 50 μm. (**C**) Western blot analysis of GmPHD3-EGFP fusion protein using the anti-GFP antibody. The asterisk indicates the expected full-length fusion protein (~64 kDa) in the GmPHD3-EGFP sample. No free EGFP (~27 kDa) was detected in this lane. The free EGFP control shows a band at ~27 kDa. Ponceau S staining served as a loading control.

To clarify the subcellular localization of GmPHD3, we constructed the recombinant expression vector PR101-*GmPHD3*-EGFP (Figure 3A). The vector was co-expressed with the nuclear marker H2B-RFP in leaves of Nicotiana benthamiana via Agrobacterium-mediated transient infiltration. Laser confocal microscopy revealed that the green fluorescent signal of the PR101-GmPHD3-EGFP fusion protein partially overlapped with the red fluorescent signal of H2B-RFP (Figure 3B), indicating that GmPHD3 was predominantly localized to the nucleus.

To rule out the possibility that the observed fluorescence derived from degraded free EGFP rather than the full-length fusion protein, we performed Western blot analysis using an anti-GFP antibody (Figure 3C). A major specific band consistent with the expected molecular weight of the full-length fusion protein (approximately 70 kDa) was detected in the GmPHD3-EGFP sample. In addition, a weaker secondary band of approximately 55 kDa was also observed, which was larger than free EGFP (~27 kDa) but smaller than the full-length fusion protein. This additional band most likely represents a partial degradation product of the GmPHD3-EGFP fusion protein during protein extraction, as PHD-type transcription factors are often subject to instability or proteolytic cleavage during in vitro protein preparation, and no free EGFP band (~27 kDa) was observed in the GmPHD3-EGFP sample. Combined with bioinformatic prediction of subcellular localization, these results confirm that GmPHD3 is a nuclear-localized protein, which is consistent with its identity as a transcription factor. The presence of the degradation product does not affect this conclusion, as the full-length fusion protein remains the predominant band, and the GFP fluorescence signal further supports the nuclear localization of GmPHD3.

### 2.2. Expression Pattern Analysis of the GmPHD3 Gene

**Figure 4:**
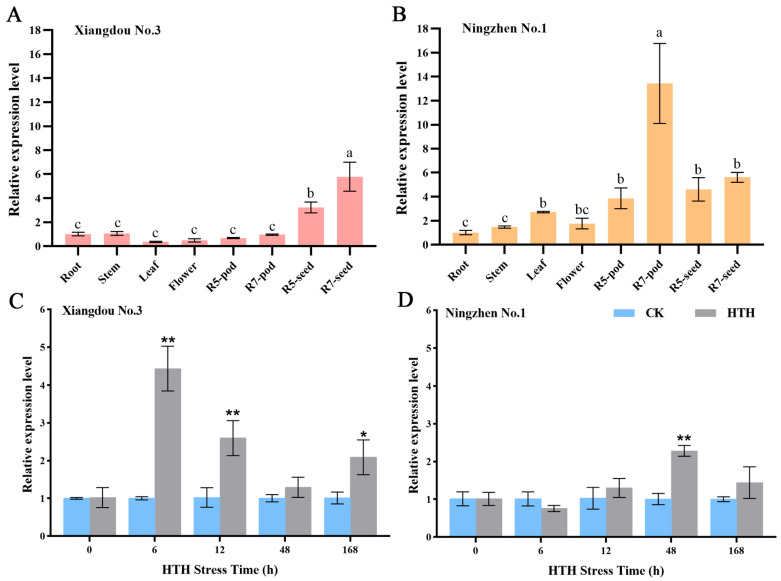
Tissue-specific and stress-induced expression of *GmPHD3* in soybean. (**A**,**B**) Expression in different tissues/organs of Xiangdou No. 3 (**A**) and Ningzhen No. 1 (**B**), including root, stem, leaf, flower, and pods/seeds at the R5 (beginning seed) and R7 (physiological maturity) stages. (**C**,**D**) Expression in response to high temperature and high humidity (HTH) stress in R7-stage seeds of Xiangdou No. 3 (**C**) and Ningzhen No. 1 (**D**). Plants at the R7 stage were treated with HTH (42 °C/30 °C, 16 h light/8 h dark, 100%/60% relative humidity) for 3 consecutive days, with samples collected at 0–168 h. *GmActin* served as the internal control. Different letters indicate significant differences among different tissues (*p* < 0.05). * indicates significant difference at *p* < 0.05, ** indicates significant difference at *p* < 0.01. CK, control; HTH, high temperature and high humidity stress.

Tissue-specific expression analysis showed that RT-qPCR results revealed significant cultivar-specific and spatiotemporal differences in *GmPHD3* transcription. In Xiangdou No. 3 (Figure 4A), *GmPHD3* was highly expressed in R7-stage seeds, with levels significantly higher than in all other tissues; in contrast, expression remained low in roots, stems, leaves, flowers, R5 pods, and R7 pods, indicating strong seed-specific expression. In Ningzhen No. 1 (Figure 4B), the highest transcript abundance was observed in R7 pods, followed by R7 seeds and R5 seeds, with lower expression in R5 pods and the lowest levels in roots, stems, leaves, and flowers. These contrasting tissue-specific expression patterns suggest that *GmPHD3* exhibits a seed-preferential expression in the HTH-tolerant cultivar Xiangdou No. 3, whereas it is most highly expressed in pods in the sensitive cultivar Ningzhen No. 1. These differences may underlie the distinct stress tolerance and seed vigor phenotypes between the two cultivars.

We further analyzed the expression patterns of *GmPHD3* under high temperature and high humidity (HTH) stress. Under control conditions, *GmPHD3* transcript levels remained low and stable without obvious fluctuations in both cultivars. Under HTH stress, *GmPHD3* expression was induced in both cultivars, but the induction patterns differed substantially. In Xiangdou No. 3 (Figure 4C), *GmPHD3* expression increased rapidly after HTH stress treatment, rising to a significant level at 6 h, remaining significantly upregulated at 12 h, decreasing to a non-significant level at 48 h, and then rising again to a significant level at 168 h, compared with the corresponding controls, indicating high sensitivity to HTH stress. In Ningzhen No. 1 (Figure 4D), *GmPHD3* expression was also upregulated by HTH stress but showed a delayed response: a mild and non-significant increase at 12 h, a continuous rise from 12 to 48 h, reaching a highly significant level at 48 h, and remaining elevated at 168 h, compared with the corresponding controls. Comparative analysis demonstrated that *GmPHD3* responded more rapidly in Xiangdou No. 3 (significant at 6 h) but showed delayed induction in Ningzhen No. 1 (significant at 48 h). In Xiangdou No. 3, expression peaked at 6 h and then declined, while in Ningzhen No. 1, the highest expression level was observed at 48 h. This difference suggests that the rapid response of *GmPHD3* in Xiangdou No. 3 may contribute to its superior stress tolerance, whereas the delayed induction in Ningzhen No. 1 may weaken the early defense ability against HTH stress.

### 2.3. Identification of Cis-Acting Elements in the GmPHD3 Promoter and Functional Validation by Transient Expression

**Figure 5:**
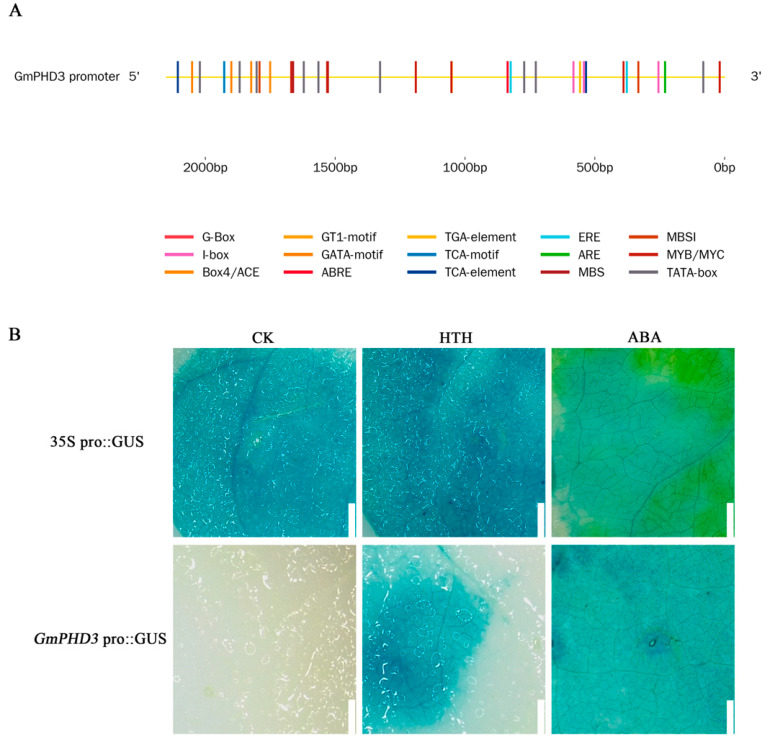
Promoter architecture and stress-inducible activity of the *GmPHD3* promoter. (**A**) Schematic diagram of the *cis*-acting regulatory elements distributed across the 2000 bp upstream region of *GmPHD3*. Putative elements include light-responsive (G-Box, GT1-motif, I-box, GATA-motif, Box4/ACE), hormone/ABA-responsive (TGA-element, ABRE, TCA-motif, ARE), stress-related (ERE, MBS, MBSI, MYB/MYC), and basal promoter (TATA-box) motifs. (**B**) Transient expression analysis of *GmPHD3* pro::GUS in tobacco leaves. Leaf samples were subjected to CK (control), HTH (3 h at high temperature and high humidity), and ABA (10 μM abscisic acid, 3 h). GUS staining intensity reflects promoter activity under each condition. Scale bar = 0.5 cm.

The *GmPHD3* promoter sequences cloned from Xiangdou No. 3 and Ningzhen No. 1 were subjected to *cis*-acting elements analysis using the Plant CARE database. The results showed that the promoter harbors multiple hormone-responsive elements, including elements responsive to salicylic acid, methyl jasmonate, auxin, and abscisic acid (ABA), as well as *cis*-acting elements associated with light signaling and drought stress (Table 1 and Figure 5A), implying that *GmPHD3* may participate in abiotic stress responses and ABA signaling regulation.

To further verify the function of this promoter, GUS histochemical staining intensity was detected in tobacco leaves after 3 h of HTH treatment or 10 μM ABA treatment. Compared with the control group, HTH treatment significantly enhanced GUS activity, and ABA treatment also increased GUS staining to a certain extent (Figure 5B).

### 2.4. Effect of GmPHD3 Overexpression on Soybean Seed Morphology and Quality Under High-Temperature and High-Humidity Stress

Wild-type Williams 82 (WM82) and T2-generation positive *GmPHD3* overexpression soybean plants were treated with HTH stress at the R7 stage. After natural ripening, T3 seeds were harvested and air-dried. Plump, intact and disease-free mature seeds were randomly selected from three overexpression lines for phenotypic observation, statistical analysis, and TTC staining analysis to explore the effects of *GmPHD3* overexpression on soybean seed morphology and quality under HTH stress.

**Figure 6:**
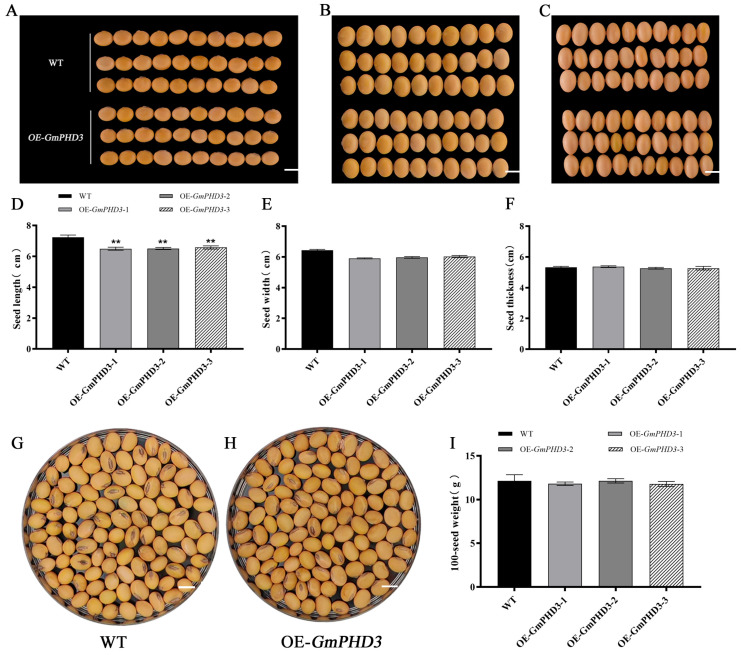
Effect of *GmPHD3* overexpression on the morphology and size of soybean seeds harvested from plants subjected to high temperature and high humidity (HTH) stress. (**A**–**C**) Morphological comparison of mature seeds from wild-type (WT) and three independent *GmPHD3* overexpressing (OE-*GmPHD3*) lines. (**D**–**F**) Statistical analysis of seed length, width, and thickness. (**G**,**H**) Phenotypic comparison of 100 randomly selected mature seeds from WT and OE-*GmPHD3* plants. (**I**) Statistical analysis of 100-seed weight. All seeds were randomly selected from plants subjected to HTH stress (42 °C/30 °C, 16 h light/8 h dark photoperiod, 100%/60% relative humidity) at the physiological maturity (R7) stage, followed by natural maturation in the field. Scale bar: 1 cm. ** indicates significant difference at *p <* 0.01.

The results showed that compared with the wild type (WT), the three *GmPHD3* overexpression lines (OE-*GmPHD3*-1, OE-*GmPHD3*-2, OE-*GmPHD3*-3) exhibited significantly reduced seed length (*p* < 0.01), whereas seed width and 100-seed weight changed slightly but did not reach statistical significance. Specifically, the average ten-seed length of WT was approximately 7.5 cm, while that of the three overexpression lines decreased to 6.5–7.0 cm (Figure 6A,D). For seed width, the WT value was about 6.5 cm and those of the overexpression lines were about 6.0 cm (Figure 6B,E). For seed thickness, the WT value was about 5.5 cm and those of the overexpression lines were about 5.8 cm (Figure 6C,F). For 100-seed weight, WT was approximately 12.5 g and the overexpression lines ranged from 12.0 to 12.5 g, showing only small fluctuations (Figure 6G–I).

**Figure 7:**
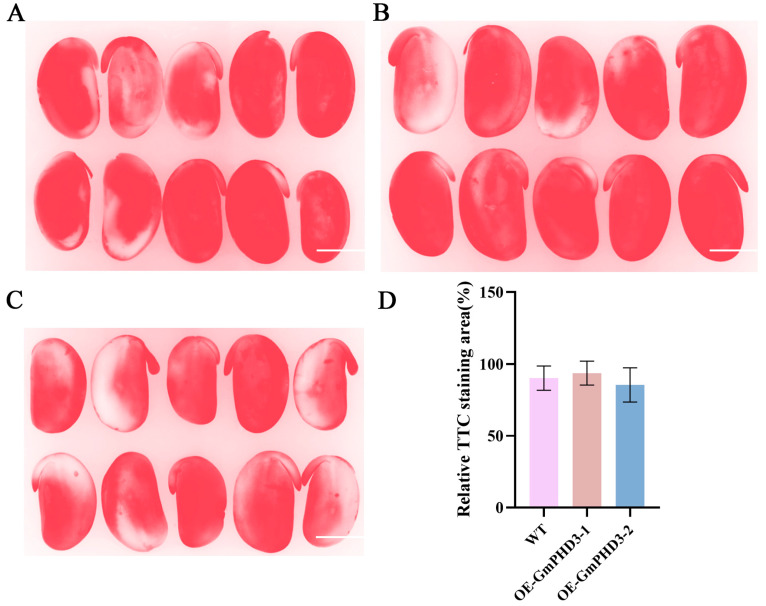
Comparison of seed viability between wild-type (WT) and *GmPHD3* overexpressing soybean seeds without HTH stress. (**A**–**C**) TTC staining results of mature seeds from WT (**A**), *GmPHD3* overexpressing-1 (**B**) plants, and *GmPHD3* overexpressing-2 (**C**) plants. All seeds were randomly selected from plants. Prior to staining, seeds were pre-germinated for 16 h, followed by TTC staining for 12 h. Scale bar = 1 cm. (**D**) ANOVA analysis of the TTC-stained area between the WT and *GmPHD3* overexpressing lines.

To investigate the effect of *GmPHD3* overexpression on soybean seed vigor, TTC staining analysis was performed in this study. Mature seeds of the wild type (WT, Figure 7A) and *GmPHD3*-overexpressing lines (OE-*GmPHD3*-1, OE-*GmPHD3*-2, Figure 7B,C) were selected for staining treatment. TTC can be reduced to red formazan by active dehydrogenases in living cells, and the staining intensity directly reflects the respiratory activity and vigor level of seeds. The results showed that seeds of WT and the two overexpression lines were uniformly stained red, and there was no significant difference in the relative TTC staining area quantified by ImageJ (1.8.0_172) (Figure 7D), indicating that *GmPHD3* overexpression did not significantly alter the basic dehydrogenase activity and overall vigor level of seeds.

### 2.5. Effect of GmPHD3 Overexpression on Soybean Seed Quality Under High-Temperature and High-Humidity Stress

Naturally dried soybean seeds previously exposed to HTH stress were used in this section. Approximately 100 seeds from the wild-type (WT) and *GmPHD3* overexpression lines (OE-*GmPHD3*) were randomly selected with three biological replicates, and seed quality traits were systematically analyzed using near-infrared non-destructive detection, including main nutritional components, fatty acid composition, and amino acid composition.

**Figure 8:**
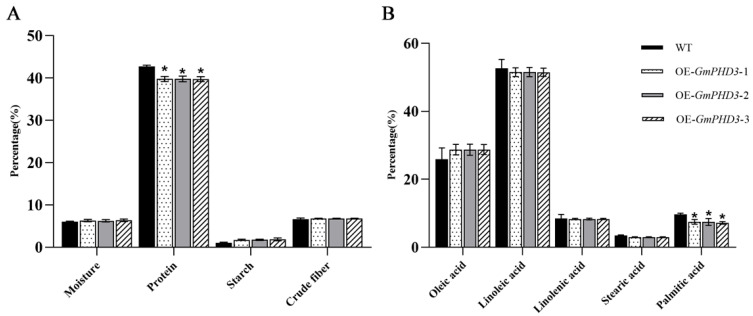
Effect of *GmPHD3* overexpression on the quality of mature soybean seeds harvested after high temperature and high humidity (HTH) stress. All seeds used in the analysis were randomly selected mature dry seeds from wild-type (WT) and three independent *GmPHD3* overexpressing (OE-*GmPHD3*) lines, which were subjected to HTH stress (42 °C/30 °C, 16 h light/8 h dark photoperiod, 100%/60% relative humidity) at the physiological maturity (R7) stage and then naturally matured in the field. For each sample, approximately 100 seeds were used, with three technical replicates performed. (**A**) Analysis of the main nutritional components (moisture, protein, starch, and crude fiber) in seeds. (**B**) Analysis of the fatty acid composition (oleic acid, linoleic acid, linolenic acid, stearic acid, and palmitic acid) in seeds. * indicates significant difference at *p* < 0.05.

The results showed no significant differences in moisture, crude fiber, or total oil content between the WT and OE-*GmPHD3* seeds. However, protein content was significantly lower in the OE-*GmPHD3* seeds than in the WT seeds (*p <* 0.01), whereas starch content showed an increasing trend without statistical significance, suggesting that *GmPHD3* overexpression changes the carbon–nitrogen allocation pattern by promoting starch accumulation and repressing protein synthesis (Figure 8A).

For fatty acid composition, no significant differences were observed in the relative contents of oleic acid, linoleic acid, linolenic acid, and stearic acid between the two genotypes. Notably, the relative content of palmitic acid, a major saturated fatty acid, was significantly lower in the OE-*GmPHD3* seeds than in the WT seeds (*p <* 0.05), indicating that *GmPHD3* overexpression optimizes fatty acid composition and reduces the proportion of saturated fatty acids without influencing the total oil content (Figure 8B).

### 2.6. Effect of GmPHD3 Overexpression on Soybean Seed Vigor Under High Temperature and High Humidity Stress

To investigate the regulatory role of *GmPHD3* overexpression in soybean seed germination and stress resistance, wild-type WM82 and T2-generation positive *GmPHD3* overexpressing plants were treated with HTH stress at the R7 stage. T3 seeds were harvested and subjected to germination assays under normal (Mock) and artificial accelerated aging conditions. We recorded the dynamic changes in germination percentage, and calculated the germination index and vigor index to compare the germination capacity and stress tolerance of the two genotypes.

**Figure 9:**
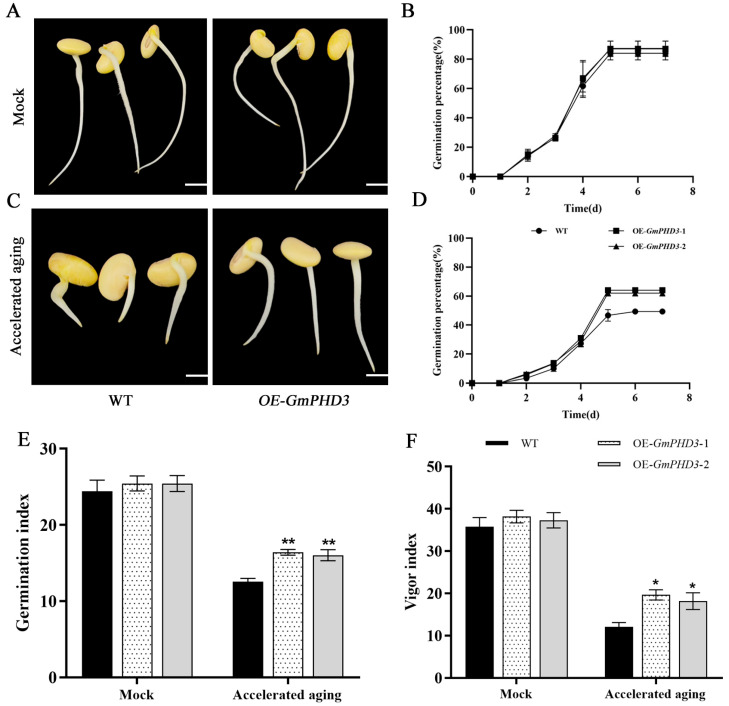
Effect of *GmPHD3* overexpression on soybean seed vigor after high temperature and high humidity (HTH) stress. (**A**,**B**) Germination phenotypes and time-course germination rates of seeds under mock control conditions. (**C**,**D**) Germination phenotypes and time-course germination rates of seeds after accelerated aging treatment. (**A**,**C**) Representative seedling phenotypes were photographed after 7 days of germination in the dark. (**E**,**F**) Germination index (**E**) and vigor index (**F**) of seeds in the mock control and accelerated aging groups. WT: wild-type; OE-*GmPHD3*: *GmPHD3* overexpressing lines. * indicates significant difference at *p <* 0.05, ** indicates significant difference at *p* < 0.01. Scale bar: 1 cm.

Under normal conditions, the WT and OE-*GmPHD3* seeds showed highly consistent germination performance: the germination percentage exhibited a similar trend, and no significant differences were detected in final germination percentage, germination index, or vigor index, except for a slight increase in the germination rate at the middle stage in the overexpression lines (Figure 9A,B,E,F). These results indicate that *GmPHD3* overexpression does not negatively affect basal germination ability or normal seedling growth, but slightly promotes the germination process.

Under accelerated aging treatment, germination performance differed significantly between genotypes. OE-*GmPHD3* seeds initiated germination earlier, displayed a distinct advantage at the early germination stage, maintained a higher germination rate throughout the entire period, and showed significantly higher final germination percentage (Figure 9C,D), germination index, and vigor index than the WT seeds, suggesting stronger germination ability, better uniformity, and higher overall seed vigor (Figure 9E,F).

### 2.7. Effects of GmPHD3 Overexpression on Antioxidant Enzyme Activities and TBARS Content in Soybean Seeds Under High Temperature and High Humidity Stress

Wild-type WM82 and T2-generation positive *GmPHD3*-overexpressing soybean plants were treated with HTH stress for 3 days at the R7 developmental stage. The harvested T3 generation seeds were used to determine the activities of superoxide dismutase (SOD), peroxidase (POD), and catalase (CAT), as well as thiobarbituric acid reactive substances (TBARS) content under normal (Mock) and artificial accelerated aging conditions, so as to systematically compare the antioxidant capacity between the two genotypes.

**Figure 10:**
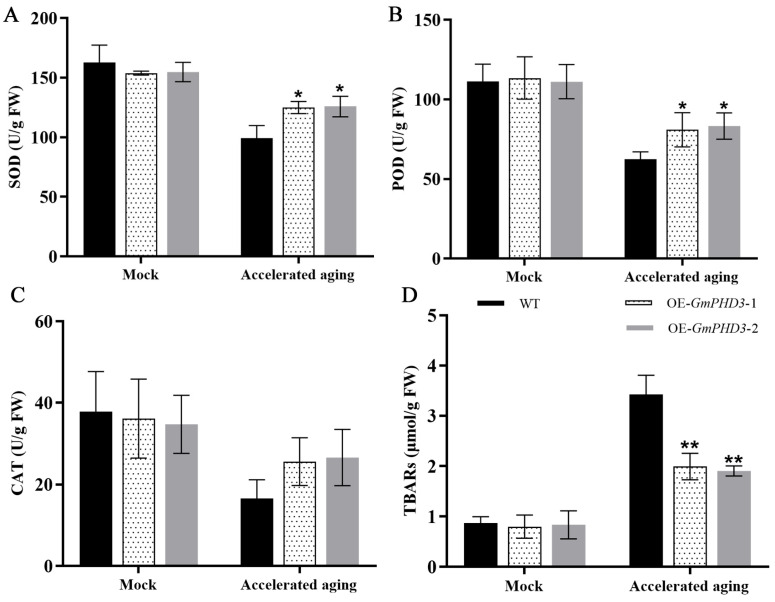
Effect of *GmPHD3* overexpression on antioxidant enzyme activities and thiobarbituric acid reactive substances (TBARS) content in soybean seeds after high temperature and high humidity (HTH) stress. All seeds used were naturally matured seeds harvested from plants subjected to HTH stress in the field. Seeds were subjected to either mock treatment (ddH_2_O) or artificial accelerated aging, then germinated for 7 days. The axes of plumules were collected for the following assays: (**A**) superoxide dismutase (SOD) activity; (**B**) peroxidase (POD) activity; (**C**) catalase (CAT) activity; (**D**) TBARS content. For each of the three independent *GmPHD3* overexpressing lines and the wild-type (WT) control, approximately 10 seeds were randomly selected, with three technical replicates performed. * indicates significant difference at *p* < 0.05, ** indicates significant difference at *p* < 0.01.

Under normal growth conditions, WT and OE-*GmPHD3* seeds showed no obvious differences in SOD, POD, and CAT activities, and maintained a low TBARS level without significant divergence. These results suggest that *GmPHD3* overexpression cannot change the basic antioxidant enzyme activities and endogenous membrane lipid peroxidation level of soybean seeds (Figure 10A–D). After artificial accelerated aging stress, distinct differences were observed in the antioxidant system between the two genotypes. Compared with wild-type seeds, OE-*GmPHD3* seeds possessed significantly higher SOD and POD activities (*p* < 0.05) (Figure 10A,B), accompanied by increased CAT activity (Figure 10C), which jointly strengthened the reactive oxygen species (ROS) scavenging system. In addition, TBARS content rose sharply in wild-type seeds after stress treatment, whereas it was extremely significantly decreased in *GmPHD3* overexpression seeds (*p <* 0.01) (Figure 10D), demonstrating alleviated membrane lipid peroxidation and better maintenance of cell membrane integrity in overexpression materials.

## 3. Discussion

In this study, both the coding sequence and promoter sequence of *GmPHD3* were completely identical between the HTH-tolerant cultivar Xiangdou No. 3 and the HTH-susceptible cultivar Ningzhen No. 1, indicating high sequence conservation of both the coding and promoter regions of *GmPHD3* in different soybean genotypes. This result is consistent with previous genome-wide studies demonstrating that most soybean gene families exhibit high intraspecies sequence homology [20,21,22]. The GmPHD family analyzed in the present study also conforms to this conservative characteristic, with most members showing highly conserved protein sequences across varieties. Similarly, PHD homologs in Arabidopsis [23] and other plant species are structurally conserved among diverse germplasms, implying that the intact protein architecture of PHD family proteins is essential for maintaining their fundamental biological functions.

Further domain analysis showed that GmPHD3 harbors two typical conserved functional domains: an N-terminal Alfin domain that specifically recognizes DNA *cis*-acting elements and represents a hallmark of ALFIN-like transcription factors [4,24], and a C-terminal PHD-AL plant zinc-finger domain containing conserved zinc-binding and histone H3-binding sites. This structural feature suggests that GmPHD3 may participate in chromatin remodeling and epigenetic modulation by recognizing histone modifications such as H3K4me3 [25]. This dual-domain architecture is also conserved in ALFIN-like proteins in turnip [26]. Consistent with previous reports in other plant species [27], the conserved structural composition of GmPHD3 confirms that it possesses the canonical structural basis of functional PHD transcription factors [16,23]. Collectively, the high sequence conservation and complete dual-domain composition of GmPHD3 provide a solid molecular foundation for its function in regulating soybean seed vigor.

PHD family proteins are well-characterized regulators of plant abiotic stress tolerance and actively participate in responses to high temperature, drought, and salinity stresses [18]. For instance, *MtPHD6* from Medicago truncatula is drought-inducible and enhances drought tolerance in transgenic Arabidopsis [28]. Similarly, *ThPHD5* from Tamarix hispida is significantly upregulated by salt, PEG, and ABA treatments [29], and several *GarPHD* genes in Gossypium aridum respond to salt, drought, and low-temperature stresses [30]. In agreement with these findings, our results demonstrated that *GmPHD3* is significantly induced by HTH stress in both soybean cultivars, consistent with the general stress-responsive pattern of plant *PHD* genes. Notably, *GmPHD3* exhibited distinct spatiotemporal expression patterns between tolerant and susceptible cultivars: rapid induction and peak expression at 6 h in Xiangdou No. 3, whereas delayed and prolonged expression peaking at 48 h in Ningzhen No. 1. This discrepancy indicates that the early and rapid transcriptional response of *GmPHD3* is closely associated with soybean HTH tolerance. In addition, tissue-specific expression analysis revealed preferential high expression of *GmPHD3* in physiologically mature seeds of the tolerant cultivar but in pods of the susceptible cultivar, further supporting its critical role in seed vigor regulation. Promoter sequence analysis identified multiple hormone and stress-related *cis*-acting elements in the *GmPHD3* promoter, consistent with its positive response to HTH treatment. Further sequence alignment of the ORF and promoter regions of *GmPHD3* between Xiangdou No. 3 and Ningzhen No. 1 revealed that they are essentially identical in both cultivars, suggesting that the differential expression of this gene is not attributable to variations in the *cis*-acting elements of the gene itself between cultivars. Notably, although GmPHD3 functions as a PHD-type transcription factor, its own transcription still requires activation by upstream regulatory factors. Therefore, we speculate that the rapid induction of *GmPHD3* in Xiangdou No. 3 is primarily attributable to cultivar-specific differences in its upstream transcriptional regulatory network; that is, the HTH stress signal can more efficiently activate the expression or activity of certain positive transcription factors in the tolerant cultivar, thereby rapidly initiating *GmPHD3* transcription, whereas in the susceptible cultivar, the signal transduction efficiency of this pathway is lower, resulting in delayed induction. Given that GmPHD3 itself is a transcriptional regulator, changes in its expression level would in turn affect the transcription of its downstream target genes, ultimately mediating the differential stress tolerance between cultivars. These results indicate that *GmPHD3* enhances soybean HTH tolerance by perceiving and responding to HTH stress signals.

PHD proteins widely regulate plant vegetative and reproductive development [9,12]. In Arabidopsis, the PHD protein VIN3 regulates vernalization and flowering transition by mediating H3K9 and H3K27 methylation of the floral repressor FLC [31]. In pepper, *CaPHD17* shows differential expression between sterile and maintainer lines during early floral development, suggesting its involvement in male fertility regulation [32]. Consistent with these studies, our results demonstrated that *GmPHD3* participates in soybean seed development and germination. Importantly, we identified a novel function of *GmPHD3* in modulating soybean seed quality: overexpression of *GmPHD3* significantly reduces palmitic acid content, providing a new candidate gene for soybean quality improvement. In terms of stress resistance, *GmPHD3* overexpression effectively alleviates HTH-induced seed deterioration by increasing SOD and POD activities, improving ROS scavenging capacity, reducing TBARS accumulation, and mitigating membrane lipid peroxidation, thereby improving seed germination performance under HTH stress. These findings provide solid physiological and molecular evidence for the mechanism by which *GmPHD3* positively regulates soybean seed vigor under HTH stress.

Although this study verified that *GmPHD3* improves soybean seed vigor and plant tolerance to HTH stress, it still has several limitations. We only clarified its physiological functions and preliminary molecular regulatory effects. Its upstream signaling regulators and direct downstream target genes have not been identified. The core downstream functional genes for *GmPHD3*-mediated HTH stress tolerance also remain unknown, which prevents a full understanding of its molecular regulatory mechanism. Nevertheless, based on the observed phenotypic changes upon *GmPHD3* overexpression, reduced palmitic acid content and enhanced SOD/POD activities, we speculate that *GmPHD3* may regulate downstream genes involved in fatty acid metabolism (e.g., genes encoding fatty acyl-ACP thioesterases) and ROS scavenging pathways (e.g., peroxidase-encoding genes). This speculation is supported by studies on homologous PHD proteins in soybean: GmPHD2 upregulates eight stress-responsive genes including peroxidases; GmPHD5 binds to promoters of stress-inducible genes such as *GmRD22* and *GmGST*; and GmPHD6 directly activates *CYP82C4*, *CYP75B1*, and *CCD7* to enhance stress tolerance [19]. These findings suggest that GmPHD3 may similarly regulate a suite of downstream effectors involved in lipid metabolism and antioxidant defense, although direct target identification awaits future investigation. In future research, we will use yeast one-hybrid and yeast two-hybrid assays to analyze the regulatory network of *GmPHD3*. We will also apply mass spectrometry-based proteomics to screen differentially expressed proteins induced by HTH stress in tolerant soybean varieties. This helps identify key downstream genes regulated by *GmPHD3* and complete the relevant molecular pathway. Furthermore, the overexpression experiments in this study were performed in the cultivar WM82, which was chosen primarily for its high transformation efficiency and well-established regeneration system rather than for its intrinsic stress tolerance. Therefore, the phenotypic effects observed in WM82 may not fully represent the functional efficacy of *GmPHD3* in a truly sensitive genetic background. To provide more direct genetic evidence, our subsequent studies will focus on generating stable *GmPHD3* overexpressing transgenic lines in Ningzhen No. 1, a cultivar known to be sensitive to combined HTH stress. These experiments will directly test whether *GmPHD3* can functionally complement the susceptibility of Ningzhen No. 1 under HTH stress, thereby strengthening the translational value of our findings for soybean molecular breeding. In addition, gene function was only verified with overexpression lines under laboratory simulated conditions. No gene knockout tests or field experiments were conducted. We will generate loss-of-function mutants and perform field phenotypic analysis to fully confirm the biological functions and breeding value of *GmPHD3*. In agricultural production, *GmPHD3* can be used as a superior candidate gene for soybean molecular marker-assisted breeding. It can enhance seed storability and HTH stress tolerance and provide reliable genetic resources for developing new soybean varieties with high seed vigor.

## 4. Materials and Methods

### 4.1. Plant Materials

The plant materials used in this study included wild type soybean (*Glycine max*) Williams 82 (WM82), the high temperature and high humidity (HTH) tolerant soybean cultivar Xiangdou No. 3, the HTH-susceptible soybean cultivar Ningzhen No. 1, and *Nicotiana benthamiana*. Soybean plants were cultivated in greenhouses and net houses at the Baima Research and Teaching Base of Nanjing Agricultural University. At the physiological maturity stage (R7), soybean plants were transferred to a growth chamber for HTH stress treatment (42 °C/30 °C, 16 h light/8 h dark photoperiod, 100%/60% relative humidity) for 3 consecutive days. After natural ripening, seeds were harvested and processed as follows: part of the seeds were stored at 4 °C for subsequent germination assays, while the remaining seeds were flash-frozen in liquid nitrogen and stored at −80 °C for other experimental analyses.

Tobacco (*Nicotiana benthamiana*) seeds were preserved in our laboratory. Tobacco plants were grown in an intelligent greenhouse under controlled conditions (25 °C, 16 h light/8 h dark photoperiod, 60% relative humidity). Healthy one-month-old tobacco plants were selected for transient transformation assays.

### 4.2. Bioinformatics Analysis of GmPHD3

The conserved domains of GmPHD3 were predicted using the NCBI Conserved Domain Database (CDD) web server (https://www.ncbi.nlm.nih.gov/Structure/cdd/wrpsb.cgi, accessed on 1 May 2026). The Clustal Omega tool available on the multiple sequence alignment web server (https://www.ebi.ac.uk/jdispatcher/msa/clustalo, accessed on 1 May 2026) was used to analyze the conserved domains of proteins. MEGA X (10.1.7) was applied to construct the phylogenetic tree. Physicochemical properties, including molecular weight and theoretical isoelectric point (pI), were analyzed with the ProtParam tool on the ExPASy platform (https://web.expasy.org/protparam/, accessed on 1 May 2026). Signal peptides and their cleavage sites were predicted using SignalP 6.0 (https://services.healthtech.dtu.dk/cgi-bin/webface2.cgi?jobid=6A3E0AF2003CF853C00569FD&wait=20, accessed on 8 May 2026) with the eukaryotic organism model. Transmembrane helices were identified using the TMHMM 2.0 online tool (https://services.healthtech.dtu.dk/service.php?TMHMM-2.0, accessed on 8 May 2026). Subcellular localization of GmPHD3 was predicted using the Plant-mPLoc web server (http://www.csbio.sjtu.edu.cn/bioinf/plant-multi/, accessed on 10 May 2026). *Cis*-acting regulatory elements in the *GmPHD3* promoter region were analyzed using the Plant CARE database (http://bioinformatics.psb.ugent.be/webtools/plantcare/html/, accessed on 15 May 2026).

### 4.3. Soybean DNA, RNA Extraction, Reverse Transcription, and cDNA Synthesis

Soybean plants at the two-leaf stage (V2) were used as experimental materials. More than three fresh leaves were randomly sampled from each plant and pooled as a biological sample. Genomic DNA (gDNA) was extracted from 0.1 g of fresh soybean leaf tissue using the cetyltrimethylammonium bromide (CTAB) method, while total RNA was isolated from the same tissue using the TRIzol reagent method.

Different reverse transcription kits were employed for cDNA synthesis based on experimental objectives. For gene cloning, full-length cDNA was synthesized using the PrimeScript RT Reagent Kit with gDNA Eraser (Accurate Biotechnology), with the extracted total RNA as the template. For reverse transcription quantitative real-time PCR analysis (RT-qPCR), short-fragment cDNA was prepared using the Evo M-MLV RT Premix Kit (containing gDNA remover). After reverse transcription, the integrity of the synthesized cDNA was verified by agarose gel electrophoresis, and the specificity of cDNA was confirmed by PCR amplification using the internal reference gene *GmActin* primers (Actin-F/Actin-R) (Table S1).

### 4.4. Reverse Transcription Quantitative Real-Time PCR (RT-qPCR)

For tissue-specific expression analysis, soybean plants (Xiangdou No. 3 and Ningzhen No. 1) grown under normal conditions (25 °C, 16 h light/8 h dark photoperiod, 60% relative humidity) were sampled at different developmental stages: roots and stems at the V1 stage (first fully expanded trifoliate), leaves at the V2 stage (second fully expanded trifoliate), flowers at the R2 stage (full bloom), and pods and seeds at the R5 stage (seed filling initiation) and R7 stage (physiological maturity). Total RNA was extracted from these tissues, and *GmPHD3* expression levels were detected by RT-qPCR.

For HTH stress-induced expression pattern analysis, seeds of Xiangdou No. 3 and Ningzhen No. 1 were without stratification treatment surface-sterilized by soaking in 75% ethanol for 1 min, followed by 10% sodium hypochlorite for 10 min, and then rinsed thoroughly with sterile distilled water. Healthy, plump seeds without visible pests, diseases, or cracked seed coats were randomly selected and sown in pots containing a nutrient soil:vermiculite mixture (3:1, *v*/*v*). For each variety, three pots with three plants per pot (nine plants per variety) were used. Plants were grown in a greenhouse under controlled conditions (25 °C, 16 h light/8 h dark photoperiod, 60% relative humidity) until the R7 stage. The plants were then transferred to a growth chamber for HTH stress treatment (42 °C/30 °C, 16 h light/8 h dark photoperiod, 100%/60% relative humidity) to simulate natural HTH stress. Independent untreated control plants were maintained under the original normal growth conditions. Seeds were collected from both stressed and control plants at 0, 6, 12, 48, and 168 h post-stress initiation, randomly sampled from the three pots of each treatment, immediately flash-frozen in liquid nitrogen, and stored at −80 °C for subsequent RNA extraction.

cDNA was synthesized from the extracted RNA using the NovoScript Plus All-in-one 1st Strand cDNA Synthesis SuperMix (NovoProtein, Cat. No. E047) according to the manufacturer’s protocol. All cDNA samples were adjusted to equal concentrations and diluted 2-fold to serve as templates for RT-qPCR. Gene-specific primers for *GmPHD3* were designed using Primer Premier 5 software (Table S1). RT-qPCR reactions were performed using NovoStart Universal Fast SYBR qPCR SuperMix (NovoProtein, Cat. No. E401) on a Bio-Rad CF96 RT-qPCR system, following the manufacturer’s instructions. Each sample was analyzed with three technical replicates. The relative expression levels of *GmPHD3* were calculated using the 2^−ΔΔCT^ method.

All experiments were performed with at least three biological replicates per variety per time point. For tissue-specific expression data, statistical significance was evaluated by one-way analysis of variance (ANOVA) followed by Duncan’s multiple range test (DMRT) using SPSS software (R27.0.1.0, IBM, Armonk, NY, USA), with a significance threshold of *p* < 0.05. Different lowercase letters (a, b, c) indicate significant differences among tissues. For HTH stress time-course data comparing stressed and control samples at each time point, a two-tailed unpaired Student’s *t*-test was performed using GraphPad Prism 8.0 (GraphPad Software, San Diego, CA, USA). Significance levels are denoted as * *p* < 0.05 and ** *p* < 0.01.

### 4.5. Subcellular Localization Assay

The recombinant vector pRI101-*GmPHD3*-EGFP was constructed by restriction enzyme digestion combined with homologous recombination and transformed into *Agrobacterium tumefaciens*. The transformed Agrobacterium cultures harboring pRI101-*GmPHD3*-EGFP and the nuclear marker vector H2B-RFP were mixed and co-infiltrated into the abaxial epidermis of *Nicotiana benthamiana* leaves using a needleless syringe. After infiltration, the tobacco plants were incubated in darkness for 24 h and then cultured under normal light conditions for another 24–48 h. The EGFP and RFP fluorescence signals were observed and captured using a laser confocal scanning microscope. The subcellular localization of GmPHD3 was determined according to the co-localization pattern of green fluorescence with the nuclear red fluorescent marker.

### 4.6. Analysis of GmPHD3-EGFP Fusion Protein by Western Blot

Leaf tissues of *Nicotiana benthamiana* transiently expressing pRI101-EGFP and pRI101-GmPHD3-EGFP were collected for total protein extraction; the leaf samples were fully ground in liquid nitrogen, mixed with plant protein extraction buffer containing protease inhibitors, and all operations were conducted at low temperature to reduce protein degradation. After complete lysis, the samples were centrifuged at 12,000 rpm and 4 °C for 15 min, and the supernatant was collected as total soluble plant protein. The protein samples were blended with 5×SDS-PAGE loading buffer and heated in a boiling water bath for 10 min to achieve thorough protein denaturation. Subsequently, the denatured proteins were separated via 10% sodium dodecyl sulfate-polyacrylamide gel electrophoresis (SDS-PAGE), and then transferred from the gel onto a nitrocellulose (NC) membrane. The membrane was blocked with 5% non-fat milk at room temperature for 1 h to eliminate non-specific binding, followed by incubation with the primary anti-GFP antibody overnight at 4 °C. After thorough washing with TBST buffer the next day, the membrane was incubated with mouse secondary antibody at room temperature for 1 h. After another round of washing, specific protein bands were visualized using an enhanced chemiluminescence (ECL) imaging system, and Ponceau S staining was applied simultaneously to confirm consistent protein loading across samples. The integrity of pRI101-GmPHD3-EGFP fusion protein was verified by comparing the apparent molecular weight of detected bands with its theoretical value, and pRI101-EGFP alone was used as the negative control to guarantee the accuracy of subcellular localization results.

### 4.7. Cloning of the GmPHD3 Promoter

The approximately 2500 bp promoter sequence upstream of the *GmPHD3* start codon (ATG) was retrieved from the NCBI database. Gene-specific amplification primers were designed using Primer Premier 5 software (Table S1). The *GmPHD3* promoter regions from Xiangdou No. 3 and Ningzhen No. 1 were cloned via polymerase chain reaction (PCR) using the Apex HF HS DNA Polymerase Premix (Accurate Biotechnology), following the manufacturer’s instructions.

### 4.8. Histochemical GUS Staining of Tobacco Leaves for GmPHD3 Promoter Transient Expression

The constructed promoter recombinant plasmid and empty vector were separately transformed into Agrobacterium. After bacterial propagation, tobacco transient transformation was carried out following the methods described in the subcellular localization assay. The infiltrated tobacco leaves were treated with HTH stress (42 °C/30 °C, 16 h light/8 h dark photoperiod, 100%/60% relative humidity) for 3 h and 10 μmol ABA for 24 h before sample collection.

Collected leaf samples were fully immersed in GUS staining solution and incubated overnight in darkness for no less than 12 h. Subsequently, samples were decolorized with 95% ethanol in a 65 °C water bath for 2–3 h until all background pigment was eliminated. The decolorization solution was then discarded, and distilled water was added to maintain leaf moisture. Finally, the stained leaves were observed and photographed under a stereomicroscope with extended depth of field by adjusting background and light conditions, and images were acquired using Las-X software (3.7.121655).

### 4.9. Construction of GmPHD3 Overexpression Vector and Soybean Genetic Transformation

The full-length open reading frame (ORF) of *GmPHD3* was amplified using specific primers carrying homologous arms matching the 5941-EGFP vector (Table S1). The amplified PCR products were separated via agarose gel electrophoresis, and target fragments were purified with the Trelief Gel Extraction Kit (Tsingke Biotechnology). The recovered fragments were ligated into the linearized 5941-EGFP vector, and the recombinant constructs were subsequently transformed into Escherichia coli and Agrobacterium tumefaciens strain EHA105. Soybean seeds were surface-sterilized with a mixed solution containing 10% sodium hypochlorite and concentrated hydrochloric acid for no less than 16 h, and then placed on germination medium for pre-germination over 16 h. Soybean genetic transformation was conducted via the hypocotyl transformation method. Half-cotyledon explants with growth points were isolated and slightly wounded, then immersed in co-cultivation bacterial solution for 30–35 min. After removing excess bacterial liquid, the explants were incubated in darkness for 3 days for co-cultivation. Subsequently, the explants were transferred sequentially to induction medium, elongation medium, and rooting medium for successive tissue culture. Regenerated intact seedlings were hardened off in a greenhouse (25 °C, 16 h light/8 h dark photoperiod, 60% RH) for 2–3 days. After rinsing off residual medium and trimming redundant roots, seedlings were transplanted into pots filled with nutrient soil and vermiculite at a volume ratio of 3:1. All transplanted seedlings were covered to maintain humidity for 7 days, then cultured under conventional growth conditions for subsequent positive plant screening and seed collection.

### 4.10. TTC Staining and Relative Stained Area Analysis of Soybean Seeds

Plump, intact, and disease-free mature seeds were randomly selected from two independent overexpression lines (OE-*GmPHD3*-1 and OE-*GmPHD3*-2) and the wild-type (WT) control, with ten seeds per line used for subsequent analyses. After imbibition in distilled water at 30 °C for 16 h, seeds were longitudinally cut and incubated in 1% (*w*/*v*) TTC solution at 30 °C in the dark for 12 h. Stained seeds were photographed under a stereomicroscope with consistent settings. Images were converted to 8-bit grayscale in ImageJ, and total seed area (A_total) and red-stained viable area (A_live) were measured by freehand tracing. The relative stained area was calculated as (A_live/A_total) × 100%.

Data were analyzed by one-way ANOVA followed by LSD multiple comparison using GraphPad Prism 8.0 software, with *p* < 0.05 considered significant.

### 4.11. Determination of Soybean Seed Vigor

Standard germination test: One hundred seeds were randomly selected from each *GmPHD3* transgenic line and wild-type control, with three biological replicates. Seeds were surface-sterilized sequentially with 75% ethanol for 1 min and 10% sodium hypochlorite solution for 10 min, then dried gently and evenly placed in 90 mm Petri dishes lined with three layers of filter paper moistened with 10 mL of distilled water. All samples were incubated in a growth chamber at 25 °C with a 24 h dark photoperiod and 60% relative humidity for 7 days, and seed germination status was recorded daily. After cultivation, seedling root length was measured. The germination index (GI) was calculated as GI = ∑(Gt/t), and the vigor index (VI) was calculated as VI = GI × root length.

The artificial accelerated aging treatment was performed under alternating temperature and humidity conditions (42 °C/30 °C, 16 h light/8 h dark photoperiod, 100%/60% relative humidity) in a growth chamber.

Superoxide dismutase (SOD) activity was measured via the NBT photoreduction method, and peroxidase (POD) activity was detected using the guaiacol colorimetric method [33]. Catalase (CAT) activity was determined by the titration method [33]. Thiobarbituric acid reactive substances (TBARS) content was quantified following the thiobarbituric acid reaction method [34].

Statistical analysis was performed by one-way ANOVA with GraphPad Prism 8.0 software.

## 5. Conclusions

In summary, GmPHD3 is a structurally conserved PHD transcription factor across different soybean cultivars, possessing typical N-terminal Alfin and C-terminal PHD zinc finger domains with complete functional structural basis and nuclear localization characteristics. This gene exhibits cultivar-dependent spatiotemporal expression patterns in response to HTH stress: *GmPHD3* is rapidly and early induced in the HTH-tolerant cultivar Xiangdou No. 3 but shows delayed induction in the susceptible cultivar Ningzhen No. 1. Meanwhile, *GmPHD3* displays distinct tissue-specific expression preferences, with predominant expression in mature seeds of the tolerant cultivar but in pods of the susceptible cultivar, which is closely associated with the differential seed vigor and stress tolerance phenotypes between the two genotypes. Functional verification revealed that *GmPHD3* positively regulates soybean seed HTH tolerance and germination performance by elevating SOD and POD antioxidant enzyme activities, improving ROS scavenging capacity, and alleviating membrane lipid peroxidation damage. In addition, *GmPHD3* participates in the regulation of soybean seed morphology and quality traits. Overexpression of *GmPHD3* specifically inhibits seed longitudinal elongation to form a short-round seed shape without altering single-seed weight, indicating its direction-specific regulatory role in seed morphogenesis. Furthermore, *GmPHD3* overexpression remodels seed quality by reducing palmitic acid and protein contents while increasing starch accumulation. Promoter *cis*-acting elements analysis and GUS assay results further confirmed that *GmPHD3* responds to both HTH stress and ABA signaling, suggesting that *GmPHD3* may integrate ABA signal transduction pathways to activate downstream stress-responsive genes, thereby improving seed environmental adaptability and vigor formation. Collectively, this study clarifies the multifaceted functions of *GmPHD3* in soybean seed stress tolerance, morphological development, and quality modulation, providing a reliable candidate gene and theoretical foundation for the molecular mechanism of seed vigor regulation and high-quality, stress-tolerant soybean molecular breeding. This gene can be adopted as a key target in soybean molecular breeding to develop new varieties adapted to HTH environments. It effectively reduces seed vigor loss and quality degradation during growth and storage under unfavorable field conditions. The research outcomes also provide practical references for solving common problems in commercial soybean production and seed preservation.

## Figures and Tables

**Table 1:** Types, core sequences, and functions of *cis*-acting elements in the *GmPHD3* promoter.

*Cis*-Acting Elements	Core Sequence	Number	Category	Function
G-Box	CACGTT/TCCACATGGCA	2	Light-responsive	Light responsiveness
I-box	AAGATAAGGCT/gGATAAGGTG/cGATAAGGCG	3	Light-responsive	Light responsiveness
Box 4/ACE	ATTAAT/CTAACGTATT	2	Light-responsive	Light responsiveness
GT1-motif	GGTTAA	1	Light-responsive	Light responsiveness
GATA-motif/chs-CMA1a	GATAGGG/TTACTTAA	2	Light-responsive	Light responsiveness
ABRE	ACGTG	1	Hormone-responsive	ABA responsiveness
TGA-element	AACGAC	1	Hormone-responsive	Auxin responsiveness
CGTCA-motif/TGACG-motif	CGTCA/TGACG	2	Hormone-responsive	MeJA responsiveness
TCA-element/TCA	CCATCTTTTT/TCATCTTCAT	2	Hormone-responsive	SA responsiveness
ERE	ATTTTAAA/ATTTCATA	2	Hormone-responsive	Ethylene responsiveness
ARE	AAACCA	1	Stress-responsive	Anaerobic induction
MBS	CAACTG	2	Stress-responsive	Drought-inducibility
MBSI/AT-rich sequence	aaaAaaC(G/C)GTTA/TAAAATACT	2	MYB/MYC binding	MYB/MYC2 binding
MYB/MYC	CAACCA/TAACCA/CAACAG/CATGTG	6	MYB/MYC binding	MYB/MYC binding
TATA-box	multiple variants	9	Core promoter	Core promoter element

## Data Availability

The original contributions presented in this study are included in the article. Further inquiries can be directed to the corresponding author.

## Supplementary Materials

The following supporting information can be downloaded at: https://www.mdpi.com/article/10.3390/plants15152376/s1, Figure S1: Sequence alignment of the *GmPHD3* promoter regions amplified from Xiangdou No. 3 and Ningzhen No. 1 compared with the Williams 82 reference genome; Figure S2: Sequence alignment of the *GmPHD3* ORF amplified from Xiangdou No. 3 and Ningzhen No. 1 compared with the Williams 82 reference genome. Figure S3: Creation and identification of *GmPHD3* overexpressing soybean materials. Table S1: Primer information.

## Abbreviations

The following abbreviations are used in this manuscript:

BF	Bright Field
CAT	Catalase
CDS	Coding Sequence
CTAB	Cetyl Trimethyl Ammonium Bromide
GUS	β-Glucuronidase
HTH	High Temperature and Humidity Stress
OE	Overexpression Lines
ORF	Open Reading Frame
POD	Peroxidase
RT-qPCR	Reverse Transcription-Quantitative Real-Time PCR
SOD	Superoxide Dismutase
TBARS	Thiobarbituric Acid Reactive Substances
TTC	Triphenyl Tetrazolium Chloride
