# Supplementary material for: Positive Regulation of Soybean Seed Vigor and Quality by the Zinc Finger Transcription Factor GmPHD3 Under High-Temperature and High-Humidity Stress
Source: Plants (Basel). 2026 Aug 3;15(15):2376. doi: 10.3390/plants15152376 (PMC13472627; doi:10.3390/plants15152376)
Supplement: Supplementary file 1 [file plants-15-02376-s001.zip › plants-4382585-supplementary.pdf]

Supplementary Materials

Supplementary Figure S1. Sequence alignment of the GmPHD3 promoter regions amplified from Xiangdou No. 3 and Ningzhen No. 1 compared with the Williams 82 reference genome

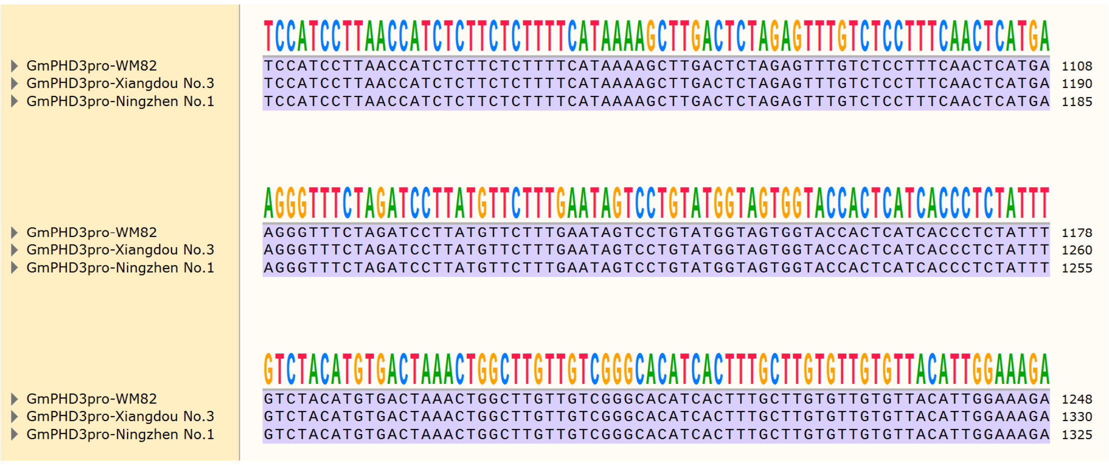

Figure S1. Sequence alignment of the *GmPHD3* promoter regions amplified from Xiangdou No. 3 and Ningzhen No. 1. Using the genomic sequence of WM82 as a reference, the promoter fragments of *GmPHD3* were PCR-amplified from the tolerant cultivar Xiangdou No. 3 and the sensitive cultivar Ningzhen No. 1, and subjected to multiple sequence alignment. The alignment revealed that the nucleotide sequences of the amplified regions are completely identical among the three cultivars, with no polymorphisms (including single nucleotide substitutions or insertions/deletions) detected. This result suggests that the differential expression of *GmPHD3* under high temperature and humidity stress between the tolerant (Xiangdou No. 3) and sensitive (Ningzhen No. 1) cultivars is not attributable to sequence variations in the core promoter region.

Supplementary Figure S2. Sequence alignment of the GmPHD3 ORF amplified from Xiangdou No.3 and Ningzhen No.1 compared with the Williams 82 reference genome

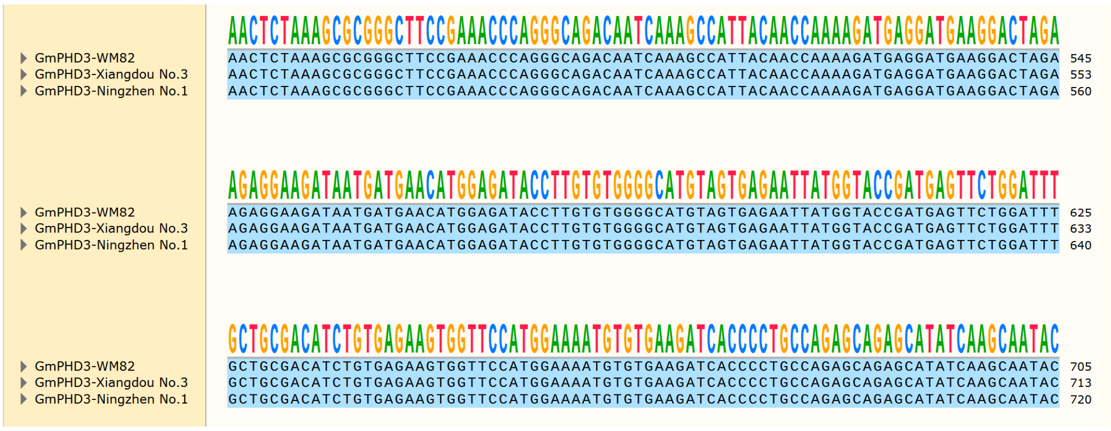

Figure S2. Sequence alignment of the *GmPHD3* ORF amplified from Xiangdou No.3 and Ningzhen No.1. Using the genomic sequence of WM82 as a reference, the ORF fragments of the *GmPHD3* gene were PCR-amplified from the tolerant cultivar Xiangdou No. 3 and the sensitive cultivar Ningzhen No. 1, and subjected to multiple sequence alignment. The alignment revealed that the nucleotide sequences of the amplified regions are completely identical among the three cultivars, with no polymorphisms (including single nucleotide substitutions or insertions/deletions) detected. This result suggests that the differential

expression of *GmPHD3* under high temperature and humidity stress between the tolerant (Xiangdou No. 3) and sensitive (Ningzhen No. 1) cultivars is not attributable to sequence variations in the ORF region.

Supplementary Figure S3. Creation and identification of GmPHD3 overexpressing soybean materials

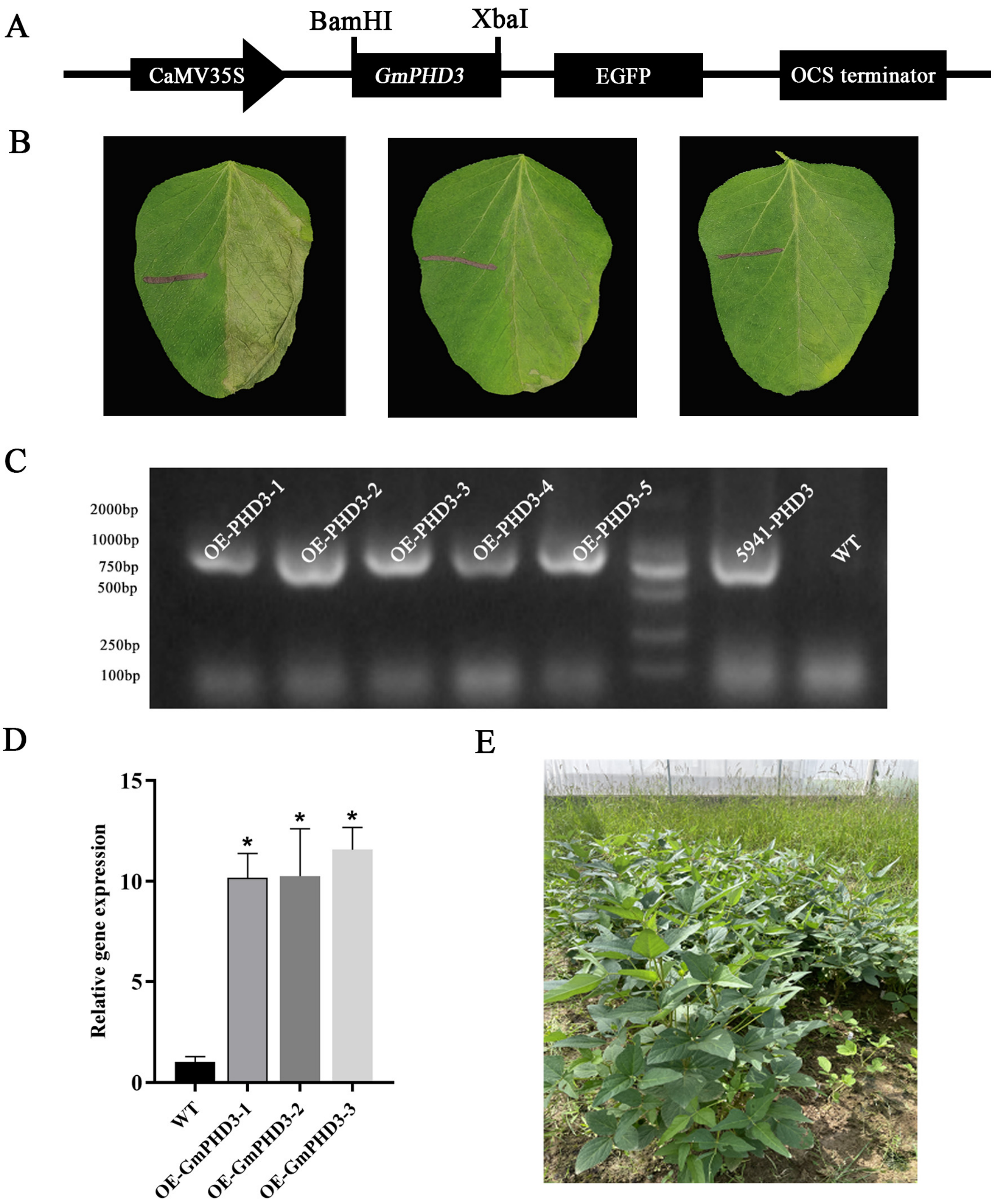

Figure S3. Generation and identification of *GmPHD3*-overexpressing soybean materials

(A) Schematic diagram of the *GmPHD3* overexpression vector driven by the CaMV35S strong promoter; (B) Phenotype of V2-stage soybean seedlings identified by glufosinate-ammonium painting, From left to right: leaf 1 (WT), leaf 2 (OE-*GmPHD3* line 1), leaf 3 (OE-*GmPHD3* line 2).; (C) PCR identification at the DNA level and sequencing validation results. M, DNA molecular weight marker; WT, wild-type control; OE, *GmPHD3*-overexpressing lines; (D) Relative expression level of *GmPHD3* detected by

qRT-PCR, * indicates $P < 0.05$; (E) Field propagation of *GmPHD3*-overexpressing lines at the Baima experimental station.

Table S1. Primer information.

Primer Name	Primer Sequence (5'- 3')	Purpose
GmPHD3-F	ATCAGGTCATTTATGGGGAGTCAGG	Gene cloning
GmPHD3-R	TCAAACATAAAAACTATCCATCAGC	
pRI101-35S-F	TGACGTAAGGGATGACGCACA	Subcellular localization
pRI101-EGFP-R	TAGGTCAGGGTGGTCACGAGG	
pRI101-GmPHD3-F	TTGATACATATGCCCGTCGACATGGAGGCGCTAAGTCGCT	
pRI101-GmPHD3-R	GCTCACCATGGATCCGGTACCGCGAGCTCTCTTGTTACTG	
*p*GmPHD3-F	GATAGCTCATGCCCAACCA	Promoter cloning and GUS staining
*p*GmPHD3-R	ATCCAACCCACTTCACCTC	
3301-proPHD3-F	GACCTGCAGGCATGCAAGCTTGATAGCTCATGCCCAACCA	
3301-proPHD3-R	TTACCCTCAGATCTACCATGGATCCAACCCACTTCACCTC	
5941E-GmPHD3-F	GAGGACACGCTCGAGGGATCCATGGAGGCGCTAAGTCGCT	Gene overexpression and detection
5941E-GmPHD3-P2A-R	ATTCTTAATTAACTCAGGCCCGGGGTTTCTTCAACATCTCCTGCTT GCTTTAACAGAGAGAAGTTCGTGGCTCCGCTTCCGCGAGCTCTCTT GTTACTG	
OE-PHD3-TEST-F	CTCCTCGGATTCCATTGCC	
OE-PHD3-TEST-R	TTACTTCCCATTGCTCATT	
